# A Review of Mercury Bioavailability in Humans and Fish

**DOI:** 10.3390/ijerph14020169

**Published:** 2017-02-10

**Authors:** Mark A. Bradley, Benjamin D. Barst, Niladri Basu

**Affiliations:** 1 School of Dietetics and Human Nutrition, McGill University, Montréal, QC H3A 0G4, Canada; mark.a.bradley@mail.mcgill.ca; 2 Department of Natural Resource Sciences, McGill University, Montréal, QC H3A 0G4, Canada; benjamin.barst@mcgill.ca

**Keywords:** methylmercury, inorganic mercury, biological availability, bioaccessibility, assimilation, biological transport, seafood, cooking, gastrointestinal tract

## Abstract

To estimate human exposure to methylmercury (MeHg), risk assessors often assume 95%–100% bioavailability in their models. However, recent research suggests that assuming all, or most, of the ingested mercury (Hg) is absorbed into systemic circulation may be erroneous. The objective of this paper is to review and discuss the available state of knowledge concerning the assimilation or bioavailability of Hg in fish and humans. In fish, this meant reviewing studies on assimilation efficiency, that is the difference between ingested and excreted Hg over a given period of time. In humans, this meant reviewing studies that mostly investigated bioaccessibility (digestive processes) rather than bioavailability (cumulative digestive + absorptive processes), although studies incorporating absorption for a fuller picture of bioavailability were also included where possible. The outcome of this review shows that in a variety of organisms and experimental models that Hg bioavailability and assimilation is less than 100%. Specifically, 25 studies on fish were reviewed, and assimilation efficiencies ranged from 10% to 100% for MeHg and from 2% to 51% for Hg(II). For humans, 20 studies were reviewed with bioaccessibility estimates ranging from 2% to 100% for MeHg and 0.2% to 94% for Hg(II). The overall absorption estimates ranged from 12% to 79% for MeHg and 49% to 69% for Hg(II), and were consistently less than 100%. For both fish and humans, a number of cases are discussed in which factors (e.g., Hg source, cooking methods, nutrients) are shown to affect Hg bioavailability. The summaries presented here challenge a widely-held assumption in the Hg risk assessment field, and the paper discusses possible ways forward for the field.

## 1. Introduction

Humans and wildlife are primarily exposed to methylmercury (MeHg) through the consumption of foods with elevated Hg concentrations [1,2]. Although MeHg sources may differ for certain human populations (e.g., pilot whale for Faroese [3,4]; rice for inland Chinese [5]), seafood represents the predominant pathway of MeHg exposure for most humans [6]. It is widely acknowledged that MeHg exposure occurs worldwide [7], and that real-world exposures have the potential to affect both human [8] and ecosystem health [2]. Accordingly, the Minamata Convention on Mercury was signed to protect human and ecosystem health from anthropogenic emissions and releases of mercury (Hg) and Hg compounds.

Balancing the risks of MeHg exposure versus the benefits associated with seafood consumption is vital given that seafood is also an important source of animal protein for much of the world’s population and there are many health benefits associated with its consumption [9,10]. To estimate human exposure to MeHg, risk assessors use standard models that incorporate the concentration of MeHg in a given seafood type along with self-reported information on portion size and consumption frequency [11]. These models include a correction factor to account for the proportion of MeHg estimated to reach systemic circulation, and make the assumption that 95% to 100% of the ingested MeHg is bioavailable. However, recent research suggests that assuming all, or most, of the ingested MeHg is absorbed into systemic circulation may be erroneous. This may result in inaccurate estimations of exposure (and ultimately risk), and as such may hamper our ability to properly balance the potential risks and benefits of seafood consumption.

The objective of this paper is to review and discuss the available state of knowledge concerning the assimilation or bioavailability of MeHg in fish and humans. Given the importance of fish as a vector for MeHg exposure in humans, but also a target for MeHg poisoning themselves, we first review studies regarding Hg assimilation in fish. Next, we review the evidence of Hg bioavailability from human-based studies. In general, peer-reviewed published papers were identified which report assimilation efficiencies or bioavailability of either Hg(II), MeHg, or both forms of Hg in fish and humans following dietary exposure.

For fish, dietary Assimilation Efficiencies (AE) are generated by measuring the amount of a chemical retained by fish after dietary exposure of a known quantity of food, and represent the net result of absorption and elimination processes [12]. Dietary AEs for fish are typically calculated as the percentage of Hg(II) or MeHg retained after a specific period of exposure and/or depuration. To review the state of knowledge concerning Hg AEs in fish, two separate literature searches were performed with SCOPUS indexing service with the last search performed on 22 October 2016. The first search was performed with the following search terms:

(“mercury” OR “* Hg” OR “methylmercury” OR “* MeHg” OR “CH_3_Hg”) AND (“assimil * efficiency” OR “intestine * transfer” OR “gut” OR “uptake pathway”) AND “fish”.

The second search included the terms:

“^203^Hg” AND (“accumulation” OR “diet”) AND “fish”.

For both searches, the terms “rat”, “mice”, “bird”, “human”, “women”, “men”, and “children” were excluded. A total of 108 studies were identified for the two searches. Of these, 25 studies reported dietary AEs for Hg(II) and/or MeHg and are reviewed here.

**Figure 1:**
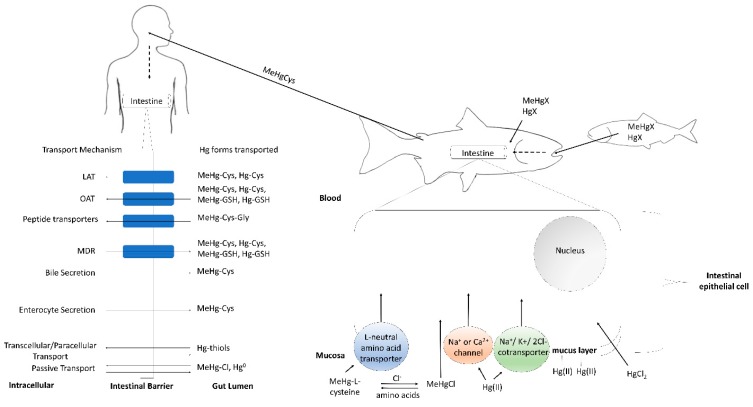
Model for methylmercury (MeHg) and inorganic mercury (Hg(II)) uptake across fish and human intestinal epithelial cells. Exposure to Hg(II) and MeHg occurs mainly through diet. Hg in both water and biological systems is bound to ligands (“X”). MeHg in fish muscle is predominately stored as MeHg-Cys [14], which enters intestinal epithelial cells through energy dependent L-type neutral amino acid transporters (LAT). MeHgCl may also enter the cell by diffusion, or by non-specific active uptake mechanisms of MeHg complexes (not shown). MeHg-Cys dominates when complexing amino acids are present [15]. Uptake of Hg(II) is presented as the model proposed by Hoyle and Handy [16]. Hg(II) may enter through voltage-gated Na^+^ or Ca^2+^ channels, through the Na^+^K^+^2Cl^−^ cotransporter, and/or by diffusion of HgCl_2_. Uptake of anionic Hg complexes (HgCl_4_^2−^) may be possible by anionic exchange (not shown). The mucus lining the gut has a high affinity for Hg(II) ions, thus limiting uptake [17]. Many of the same mechanisms occur in the human gut following MeHg ingestion through consumption of fish, with LAT playing a prominent role in transporting MeHg-Cys, while other peptide transporters and the organic anion transporter (OAT) may also contribute to uptake of MeHg-Cys (as reviewed by Bridges and Zalups [18]) Arrows are indicative of direction of transport.

For the human section, we focused on bioavailability (Figure 1; [13]) which refers to the fraction of an ingested compound that reaches systemic circulation and is thus available for biological activity. To review the state of knowledge concerning Hg bioavailability in humans, three separate literature searches were performed with SCOPUS indexing service with the last search performed on 24 October 2016. The first search was performed with the following terms:

(“mercury” OR “* Hg” OR “methylmercury” OR “* MeHg” OR “CH_3_Hg”) AND (“bioavailab *” OR “bioaccess *” OR “uptake” OR “transport” OR “assimilate *” OR “absor *”) AND “human”.

The second search included the terms:

(“mercury” OR “* Hg” OR “methylmercury” OR “* MeHg” OR “CH_3_Hg”) AND (“bioavailab *” OR “bioaccess *” OR “uptake” OR “transport” OR “assimilate *” OR “absor *”) AND “human”.

The third search included the terms:

(“mercury” OR “Methylmercury”) AND (“bioaccessibility” OR “bioavailability” OR “Caco-2”) AND (“fish” OR “seafood” OR “shellfish”).

For all searches, the terms “Soil” and “Air Pollution” were excluded. A total of 115 studies were identified for the two searches, of which 20 reported on some aspect of bioavailability for Hg(II) and/or MeHg, which were the focus of this section.

## 2. Fish

Wild fish are exposed to Hg(II) and MeHg in both water and food. Uptake of MeHg from diet accounts for approximately 80% to 90% of total uptake, with the remaining fraction coming from water [19,20]. Uptake of Hg(II) from diet (predicted by ^203^Hg radiotracer studies in tilapia) is more variable and may account for 32% to 92% of the total Hg(II) accumulation, with the remainder being absorbed from water [21]. The uptake of Hg(II) across the gills is dependent on water chemistry; increases in dissolved organic carbon (DOC) decrease uptake [22,23] while chloride concentrations that favor the production of HgCl_2_ increase absorption [23]. Though exposure to Hg(II) and MeHg via water may be more important under certain circumstances (i.e., when fish are not feeding), it is generally accepted that dietary uptake is the most important exposure pathway for fish.

Both MeHg and Hg(II) have strong binding affinities for selenium and sulfur, which within biological systems are mostly present as selenols and thiols. Cysteine (Cys) represents the most abundant protein- or peptide-bound thiol in biological systems [24], and is the major complexing agent in fish muscle tissue [14]. As a result, dietary uptake of Hg-thiol complexes in the gut is an important process which dictates the assimilation of MeHg by fish. Uptake of MeHg-l-Cys in the fish gut most likely occurs through energy-dependent large L-type neutral amino acid transporters (LAT) [15]. Uptake of MeHgCl, which predominates when cysteine and other amino acids are absent, may occur through passive diffusion and non-specific active mechanisms [15]. Further research is required to elucidate the potential non-specific active mechanisms involved in the uptake of MeHg complexes; however, transport across the intestine decreases with temperature or ouabain, an inhibitor of active transport by Na^+^, K^+^-ATPase pumps [15] (Figure 1).

After studying the absorption of Hg(II) across isolated intestines of rainbow trout, Hoyle and Handy [16] developed a tentative working model for Hg(II) transfer across the vertebrate intestine (reviewed by Kidd and Batchelar [25]). The authors suggest that uptake of Hg(II) across the intestines may enter cells through voltage-gated Ca^2+^ channels, and/or by diffusion of electroneutral complexes, such as HgCl_2_. Furthermore, uptake of anionic Hg complexes may be possible by anionic exchange. Despite these various possible uptake routes, the mucosal membrane lining the gut has been shown to serve as an excellent barrier to Hg(II), as Hg(II) ions are efficiently bound to mucus at physiological pH [17] (Figure 1). This is consistent with a study of rainbow trout (*Oncorhynchus mykiss*) gut sacs perfused with HgCl_2_ solution which demonstrated that the majority (78%–85%) of Hg(II) accumulated in intestinal mucosa rather than in underlying tissue [16]. Boudou and Ribeyre [26] reported that following 30 days dietary exposure to HgCl_2_, 36% of the relative Hg(II) burden accumulated in the posterior intestines of rainbow trout. In a separate group of rainbow trout exposed to dietary MeHgCl for 30 days, less than 3% of the relative Hg(II) burden was found in posterior intestines. Thus, significant fractions of ingested Hg may localize largely in the intestinal mucosa and in the posterior intestines of fish.

**Figure 2:**
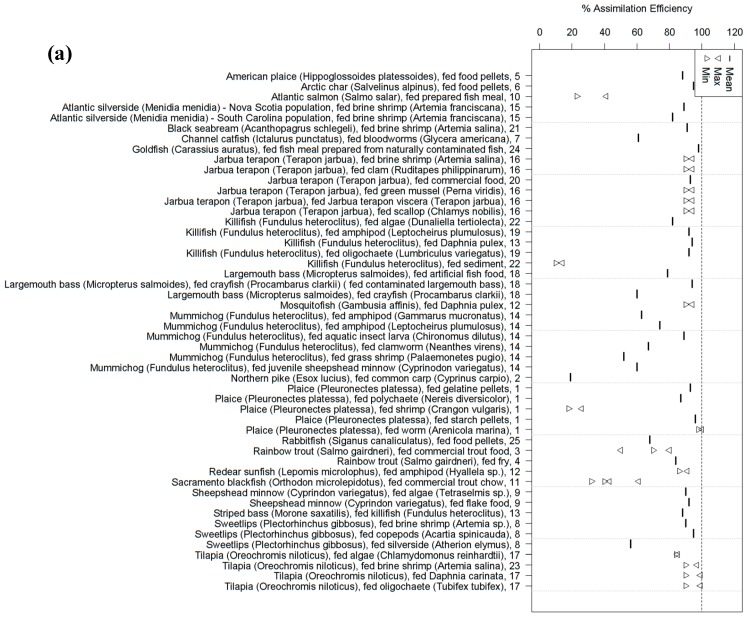

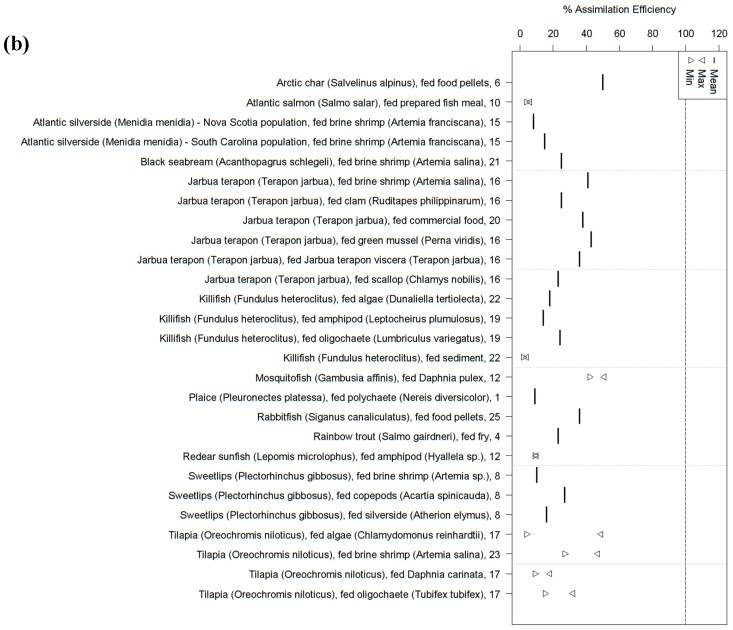
Summary of studies on AE of Hg into fish for (**a**) MeHg and (**b**) Hg(II). In both, each column represents a single species of fish, fed a single type of food. The numbers along the Y-axis indicate studies, as outlined in Table S2.

We identified 25 studies which report dietary AEs for Hg(II), MeHg, or both (Figure 2; Table S1), all conducted in vivo. Fifteen of the studies report dietary AEs for both Hg(II) and MeHg. Sixteen of the 25 studies used radioisotopes (^203^Hg and/or ^203^MeHg) as sources of Hg(II) and MeHg in their experiment(s). Seven studies used the chloride complexes HgCl_2_ and/or MeHgCl, and two studies used naturally-contaminated fish. Various food types were used to transfer Hg(II) and/or MeHg to the study fish. Nine studies report AEs from multiple food types. The tested food types included prepared fish food (including artificial and commercial foods, as well as fish meal prepared by the researchers; 11 of 25), invertebrates (13 of 25), previously exposed fish or fish parts (5 of 25), algae (3 of 25), and sediment (1 of 25). The studies ranged in duration from 36 h to 4 months in length, and reported dietary AEs for 21 different species of freshwater, marine, or euryhaline fish.

There was considerable variation among the reported dietary AEs for MeHg and Hg(II). AEs for MeHg were 10%–100% and for Hg(II) were 2%–51% (Figure 2). In general, dietary MeHg was more efficiently assimilated than Hg(II). Several of the studies indicated differences in AEs among food types. For example, sweetlips (*Plectorhinchus gibbosus*) which consumed either brine shrimp (*Artemia* sp.), copepods (*Acartia spinicauda*), or silverside (*Atherion elymus*) contaminated with Hg radioisotopes assimilated 56%–95% and 10%–27% of MeHg and Hg(II), respectively [27]. Similarly, Goto and Wallace [28] reported differences in MeHg AEs (52%–89%) after feeding mummichog (*Fundulus heteroclitus*) one of several diets of contaminated aquatic invertebrate or fish. Dutton and Fisher [29] reported low dietary AEs for Hg(II) (2%–4%) and MeHg (10%–14%) after feeding killifish spiked sediments (195 nM ^203^Hg(II) and 195 nM ^203^MeHg). As a comparison, the authors also exposed a separate group of killifish to contaminated algae (2.01 nM ^203^Hg(II) and 2.01 nM ^203^MeHg). Dietary AEs were significantly greater for both Hg(II) (18%) and MeHg (82%) from this food type, which the authors suggest was due to metal binding to more labile organic matter in algae, thus increasing the bioavailability.

There is some evidence which suggests that AEs of MeHg may be concentration and/or time dependent. After 35 days of dietary MeHg exposure, Sacramento blackfish (*Orthodon microlepidotus*) in the high-dose group (55.5 μg/g) had assimilated a significantly lower percentage of MeHg in muscle than those in the low-dose group (0.52 μg/g). However, after 75 days of exposure AEs were lower, but not significantly different among the treatment groups, which the authors suggested may have been due to decreased absorption, increased depuration, or a combination of the two [30]. In a separate study by Rodgers and Beamish [31], AE decreased from 70% to 80% to less than 50% in rainbow trout exposed to dietary MeHg (75 μg/g) for 9 weeks. In general, a decline in AE after prolonged exposure to a chemical would be expected as a dynamic steady state is reached [12].

Naturally-contaminated fish or fish muscle (that is, fish that have been contaminated with Hg in the natural environment, or muscle collected from such fish) have rarely (2 of 24 studies) been used as a source of Hg in studies reporting AEs for either MeHg or Hg(II) (Figure 2). Li et al. [32] incorporated muscle of feral fish from contaminated systems (catfish (*Ictalurus punctatus*) for high Hg exposure or walleye (*Sander vitreus*) for low Hg exposure) into food pellets for goldfish (*Carassius auratus*), and reported an AE of 98% regardless of experimental Hg exposure. Conversely, Northern pike (*Esox lucius*) assimilated only 19% of the MeHg from wild-caught common carp (*Cyprinus carpio*) used as feeder fish [33]. To our knowledge, the latter is the only study to report an AE of MeHg after using naturally-contaminated whole feeder fish. More studies that use naturally-contaminated whole feeder fish are needed in order to assess whether MeHg bioavailability is overestimated by studies using spiked diets or those using laboratory-exposed prey fish.

Following dietary exposure, MeHg quickly accumulates in the gut and then is more slowly transferred to other tissues. For example, in Arctic char (*Salvelinus alpinus*) exposed to a single dose of ^203^MeHg (0.26 ± 0.05 μg Hg/g body weight), radioactive Hg was visualized by autoradiography in the gut tract after one day, and 27 days were required for 95% of the initial dose to be transferred from the gut to blood [34]. Similarly, intestinal uptake of MeHg took place in sheepshead minnow (*Cyprinodon variegatus*) within hours of dietary exposure to MeHg (3 or 16 μg/g wet weight), and transfer to other tissues occurred more slowly, as 2 days were required for 95% of the initial dose to be transferred from intestine to blood [35].

Once in blood, MeHg associates with red blood cells and binds with hemoglobin due to its relatively high concentrations of sulfhydryl groups as observed in a study of rainbow trout [36]. In contrast to MeHg, Hg(II) binds with cysteine, albumin, and glutathione (GSH) in plasma, following absorption into blood. Blood, containing both forms of Hg, moves from the intestinal tract to the liver via the portal vein. Due to its strategic positioning within the circulatory system, the liver is exposed to dietary MeHg and Hg(II) before other tissues and thus the liver’s ability to metabolize or eliminate Hg may dictate levels in other fish tissues. The MeHg and Hg(II) remaining in blood continue to travel through the circulatory system to other parts of the body. The binding of MeHg with hemoglobin is reversible, thus facilitating MeHg transfer to other tissues [36]. In Arctic char, the transfer of MeHg from blood to tissues likely involves an intermediate passage through blood plasma, which has been suggested to be a rate-limiting step due to the low concentrations of small mobile sulfhydryl ligands in plasma [34].

Concentrations of Hg in fish are often highest in well-perfused tissues such as liver, spleen, and kidney [37,38], however muscle represents the largest pool of Hg in fish. For example, after dietary exposure to ^203^MeHg and a subsequent 30-day depuration period, 71%–77% of the accumulated MeHg was found in the muscle of tilapia (*Oreochromis niloticus*), with lower percentages found in the head (16%–18%) and viscera (5%–9%) [21]. In fish muscle tissue, >95% of the Hg(II) is present as MeHg [39]. Evidence from X-ray absorption spectroscopy has suggested that in fish muscle, MeHg is most likely bound to cysteine residues in proteins [40]. This has been substantiated by HPLC-ICP-MS analyses of dogfish muscle showing that MeHg is predominantly present as MeHgCys [14]. Therefore, MeHgCys is likely the most relevant form for human exposures as realized through the consumption of contaminated seafood muscle tissue.

Both laboratory and manipulative field studies have demonstrated that fish eliminate MeHg very slowly [41,42,43]. For example, Van Walleghen and colleagues [43] monitored Hg(II) in northern pike, which had naturally accumulated isotope-enriched MeHg through a whole-lake loading study, after their transfer to a different lake. Spiked Hg(II) was detected in muscle samples over the course of the 7-year study, and the authors estimated the half-life of Hg(II) in muscle at 3.3 years. The slow elimination of MeHg plays a role in the elevated concentrations of Hg in larger and older fish [38].

The evidence reviewed here suggests that, while the assumption that MeHg is more efficiently absorbed from the intestine than Hg(II) generally holds true, the assumption that MeHg is nearly 100% absorbed from the diet may not apply in all cases. In particular, the type of food through which fish are exposed to Hg may affect AE. It is not clear if Hg assimilation is dependent on concentration or time, though existing evidence suggests this is possible. The efficiency of MeHg transfer from food to fish, and the slow elimination of MeHg from fish, have consequences for both fish health and human health.

## 3. Humans

Humans are exposed to Hg primarily as MeHgCys from fish in the diet. The assumption that 95%–100% of the ingested MeHg is absorbed into systemic circulation is largely based on limited and outdated studies. One of the earliest studies (published in 1969) involved oral administration of aqueous methylmercuric nitrate to three middle-aged (37–44 years) Caucasian male volunteers [44]. Another early study (published in 1971) with 15 subjects (9 male, 6 female, ages 27–48, no indication of race/ethnicity) investigated Hg bioavailability after oral exposure to fish protein-bound MeHg [45]. While they are valuable as studies on humans, both suffer from limitations. Conclusions in both studies are drawn from small sample sizes. Both studies focus on acute exposures and do not utilize realistic exposure routes. For example, the studies use a relatively small portion size of ingested fish (10 g vs. the 75 g serving in Health Canada’s food guide [46] and the 85 g serving by the U.S. Food and Drug Administration (FDA) [47]), and use either aqueous methylmercuric nitrate or fish tissue spiked with methylmercuric nitrate rather than the more physiologically relevant methylmercuric cysteine, methylmercuric chloride, or MeHg bound to fish muscle tissue from natural contamination.

A body of emerging research suggests that Hg may be less than 100% bioavailable from seafood to human consumers. While these studies are beginning to explore these circumstances, there are a number of specific aspects of bioavailability that warrant much more research. As a brief primer relevant to the sections that follow, bioavailability encompasses three primary processes: bioaccessibility, absorption, and metabolism [13]. Bioaccessibility refers to the fraction of an ingested compound that is released from the food matrix into soluble form within the gastrointestinal tract; in vivo bioaccessibility represents a combined contribution of human and bacterial digestion to solubilization. Absorption refers to the movement of the bioaccessible compound into and across the intestinal epithelium to reach the blood supply; some studies break absorption up into two sub-processes: cellular retention (how much Hg accumulates inside intestinal cells) and cellular transport (how much Hg accumulates on the basolateral side of cells after introduction of Hg to the apical side). Metabolism of the compound may occur during both digestion/solubilization and absorption, which may modify the degree of bioavailability of a compound; metabolism also includes metabolism that occurs in the liver before the absorbed compound reaches the general circulation. Thus, for example, bioaccessibility can be used as a conservative estimate for bioavailability, as bioaccessibility is a theoretical maximum possible bioavailability [13,48]. Though some studies exist (and are reviewed below), surprisingly little attention has been devoted to MeHg bioavailability from food matrices in humans, though food remains the principal route of MeHg exposure in the general population.

**Figure 3:**
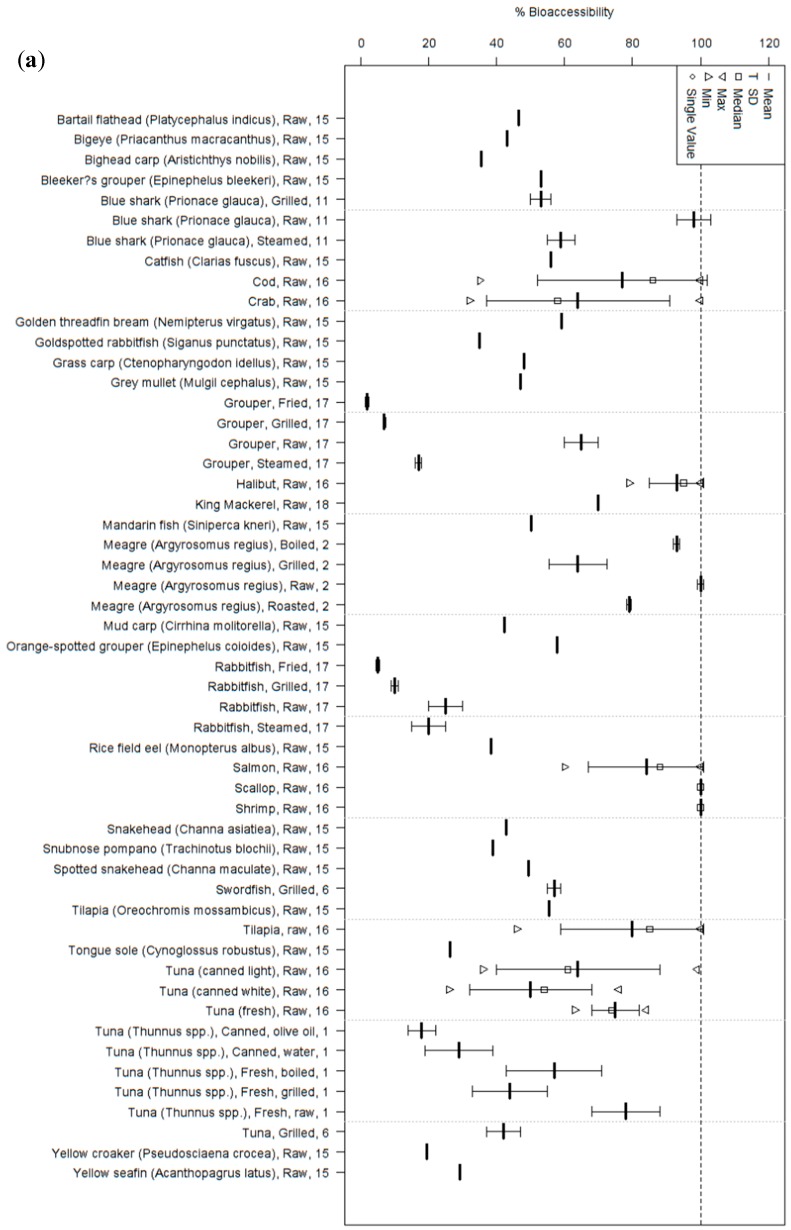

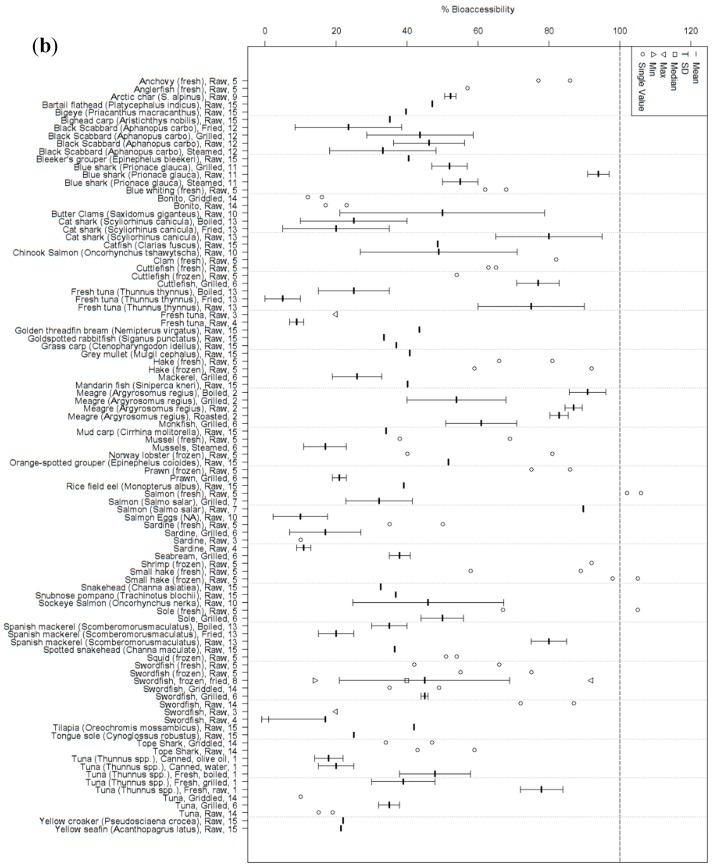
Summary of in vitro studies on bioaccessibility of Hg to humans from fish consumption for (**a**) MeHg and (**b**) total Hg. In both, each column represents a single species of fish, prepared and/or packaged in a particular way. The numbers along the Y-axis indicate studies, as outlined in Table S4.

**Figure 4:**
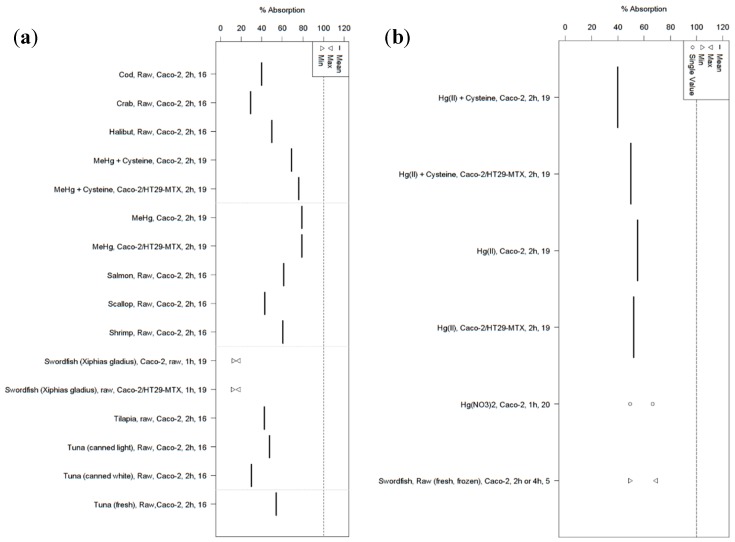
Summary of in vitro studies on absorption of Hg to human intestinal cells from fish consumption for (**a**) MeHg and (**b**) total Hg. In both, each column represents a single species of fish, prepared and/or packaged in a particular way, and introduced to either a monoculture of Caco-2 (intestinal epithelial-like cells) or a co-culture of Caco-2 and HT29-MTX (mucin-producing cells analogous to goblet cells). The numbers along the Y-axis indicate studies, as outlined in Table S4.

We identified 20 studies that report on some aspect of bioavailability (either bioaccessibility or absorption) for total Hg, MeHg, or both (Figure 3 and Figure 4; Tables S3 and S5). These studies cover 59 different types of seafood prepared in six different ways (raw, grilled, boiled, fried, steamed, or roasted) and packaged in four different ways (fresh, frozen, canned in water, or canned in oil) (note: numbers below may add up to more than the total number of studies because some studies investigated more than one aspect of bioavailability). Sixteen studies report bioaccessibility from naturally-contaminated fish, and of these fifteen report on total Hg, eight report on MeHg, and seven report on both. Only three of these in vitro studies incorporate absorption and metabolism to investigate the full process of bioavailability; two report on absorption from naturally-contaminated fish (one each reporting on total Hg and MeHg), while one reports bioavailability from a range of lab-prepared standards (reporting on both total Hg and MeHg).

Overall mean bioaccessibility estimates ranged from ~2% to 100% for MeHg and 0.2% to 94% for total Hg; overall mean absorption estimates ranged from 12% to 79% for MeHg and 49% to 69% for total Hg (Figure 3 and Figure 4; Tables S3 and S5). Some of the studies investigated only a single type of seafood (e.g., Costa et al. [49] only investigated salmon), while others investigated many types of seafood [50,51,52]. Some studies investigated only a few types of seafood, but also investigated how different ways of cooking these seafoods affected Hg bioaccessibility relative to raw samples of the same seafood [53,54].

To our knowledge, the three in vitro Hg bioaccessibility studies that have included the greatest number of seafoods have looked at 20 seafood types in Hong Kong, China [52], 16 seafood types in Valencia, Spain [50], and 10 seafood types in Montreal, Canada [51]. Since the methods used in all studies are broadly similar, examining these three studies may provide some of the clearest comparisons across different types of seafood. Though comparisons among the studies are challenged by methodological differences, at least looking within each study can provide useful comparisons between types of seafood. For example, Calatayud et al. [50] and Siedlikowski et al. [51] use near-identical methods, while Wang et al. [52] use methods that use a lower weight of fish to start the digestion and a longer small intestinal digestion (6 h vs. 2 h). In vitro total Hg bioaccessibility ranged from 21% to 52%, and MeHg bioaccessibility ranged from 20% to 59% from raw seafood in the Hong Kong study [52]. In vitro total Hg bioaccessibility from 16 raw seafood species in Spain ranged from 35% to 106%; cellular retention and transport (components of absorption, itself a component of bioavailability) after 2 h of incubation with cultured Caco-2 cells was 49%–69% and 3%–14%, respectively, in swordfish, the only seafood assayed for absorption [50]. In vitro MeHg bioaccessibility from 10 raw seafood species in Montreal ranged from 50% to 100%; absorption after 2 h of incubation with cultured Caco-2 cells ranged from 29% to 67% of the initial undigested sample [51]. Taken together, these three studies document substantial variability in Hg bioavailability from seafood, and also challenge the assumption that 95%–100% of ingested MeHg is absorbed.

Various cooking methods have been investigated for effects on Hg concentration in seafood edible tissues, and to a lesser degree bioaccessibility (Table 1). In general, cooking tends to increase the wet weight concentration of Hg in seafood, though this is almost certainly due to loss of moisture during cooking rather than any change in the amount of Hg in the seafood itself. Cooking also tends to decrease bioaccessibility of Hg from seafood, relative to raw seafood, with the more “intense” the cooking process the less bioaccessible the Hg after cooking (i.e., cooking treatments in order of most to least bioaccessible would be raw > steamed/boiled > grilled > fried). Total Hg bioaccessibility in boiled and fried fish were 40% and 60% lower, respectively, than raw samples of the same fish among samples of Spanish mackerel (*Scomberomorus maculatus*), cat shark (*Scyliorhinus* sp.) and red tuna (*Thunnus thynnus*) [54]; MeHg bioaccessibility was reduced by 75%–96% for grouper (*Epinephelus coioides*) and 29%–77% for rabbitfish (*Siganus oramin*) when steamed, grilled, or fried, compared to raw [53]. These studies concerning MeHg bioaccessibility demonstrate that cooking has an affect though most of the MeHg bioaccessibility studies have simply used raw seafood. Beyond bioaccessibility, to our knowledge no studies have investigated MeHg bioavailability from fish after different cooking methods.

Tuna has been estimated to be amongst the most relevant Hg sources worldwide [61,62,63,64,65]. Of particular note in more recent studies is that Hg from canned tuna may be less bioaccessible than Hg from raw tuna [51,66]. Afonso et al. [66] proposed that the reduction in bioaccessible Hg in canned seafood may be a result of denatured proteins becoming less accessible to the protease action and consequently less Hg is solubilized during digestion. The strong affinity of Hg for proteins [67] and the ability of protein denaturation by heat to modify the reactivity of protein-bound Hg [68] suggest it is possible that the heating involved in the canning process, similar to what is seen for various methods of cooking fish, reduces bioaccessibility of Hg from fish.

Some nutrients and foods appear to be able to modify Hg bioaccessibility. Nutrients and foods investigated for interactions with MeHg were reviewed by Chapman and Chan [69]. Examples of more recent studies on interactions between food components and MeHg from seafood are presented in Table 2. A number of notable items, including tea, coffee [54,70], and various forms of fiber (wheat bran, OAT bran, and psyllium [70]) have been associated with reducing Hg bioaccessibility in vitro. Epidemiological evidence in support is minimal, but represents a fruitful area of future research. For example, a 12-month prospective dietary assessment of 26 adult women in the Brazilian Amazon showed that those who ate more tropical fruits had lower levels of Hg in hair than those who did not, for a given number of fish meals [71]; while the researchers suggest that “absorption” (i.e., bioavailability) may be one possible mechanism explaining the finding, an in vitro study showed no effect of grapefruit juice on bioaccessibility of Hg [70].

Conflicting reports exist on associations between edible seafood tissue total Hg concentration and Hg bioavailability to humans. Some studies find that in vitro Hg bioaccessibility is independent of total Hg concentration [51,53,72,73], while others indicate a negative correlation between Hg concentration and bioaccessibility [51,73]. Both studies by Laird et al. [72,73] use the Simulated Human Intestinal Microbial Ecosystem (SHIME), one of the more complex and likely realistic models for assessing human bioaccessibility as it includes the colonic microbiota and represents a fed state. He and Wang [53] and Siedlikowski et al. [51] use simpler digestion models that represent a fasted state. Two studies have found a negative correlation between Hg concentration and cellular absorption [51,74]. Many studies that have reported on bioavailability have not explicitly investigated the relationship between concentration and bioavailability. Thus, it is not clear if MeHg concentration in edible seafood tissue may be a good proxy for MeHg bioavailability.

The emerging in vitro evidence reviewed here challenges the long-held assumption that nearly 100% of ingested Hg is absorbed into the human body by showing cases in which this assumption may not hold (i.e., cases in which bioavailability may be substantially <100%) and by highlighting limitations of some of the studies on which this assumption was based (e.g., studies using an exposure method other than naturally-contaminated fish). As discussed above for fish, it is not clear if the concentration of Hg is related to its bioavailability, though it may be.

## 4. Conclusions

There is a growing awareness that not all Hg from the diet is absorbed in both fish and in humans. The purpose of this review paper was to discuss the available state of knowledge concerning the assimilation or bioavailability of MeHg in fish and humans. In doing so, the outcome of this activity shows in a variety of organisms, experimental models, and cases that Hg bioavailability and assimilation is less than 100%. Specifically, 25 studies on fish were reviewed with AEs for MeHg ranging between 10% and 100% and for Hg(II) were 2%–51%. For humans, 20 studies were reviewed with bioaccessibility estimates ranging from ~2% to 100% for MeHg and 0.2%–94% for Hg(II); overall mean absorption estimates ranged from 12% to 79% for MeHg and 49%–69% for Hg(II) and are consistently less than 100%. The summaries presented here challenge a widely-held assumption in the Hg risk assessment field. Without being able to properly account for the true nature of Hg bioavailability and assimilation in humans and fish there will be inherent biases in assuming that the proportion of ingested Hg estimated to reach systemic circulation is 95 to 100%. Moving forward, there are several established model systems, simple to complex, available to characterize Hg bioavailability and assimilation, and when coupled with more sophisticated analytical approaches such as Hg stable isotopes, radiotracers, and speciation, the scientific community will be able to deepen our understanding of how the organism “handles” Hg; however, there is not yet an established validation method for bioavailability studies. Such knowledge is needed given that Hg remains a global contaminant of concern.

Despite growing interest on the topic there remain methodological and conceptual limitations that warrant discussion. Here we discuss some of the main limitations identified based on our review of the literature.

One challenge particular to this review is that most of the studies conducted thus far in fish have been conducted in vivo, while most of the studies focusing on humans have been conducted in vitro, and this makes it difficult to compare findings across the species. In addition, there has been a surge of recent interest in in vitro to in vivo extrapolations [75] which may prove fruitful in terms of better understanding the in vitro bioaccessibility and bioavailability studies. Despite significant mammalian research into Hg toxicity, mammalian research on Hg bioavailability is limited. In addition to there being relatively few mammalian studies on bioavailability, many of these studies do not provide clear percent values for bioavailability, but rather point to factors (e.g., dietary factors) that may increase or decrease bioavailability. As such, the majority of data comes from in vitro studies, while animal studies (and a limited number of human studies) provide context. For example, lower bioavailability from food sources artificially-spiked with Hg than from naturally-contaminated food sources has been demonstrated in both rats [76] and fish [33]. This difference deserves further consideration in discrepancies in reported Hg bioavailability between human in vivo studies [44,45] and the human-focused in vitro models that are the focus here. There are some additional challenges when it comes to studying bioavailability in humans that are not faced (or not nearly as prominent) in animal studies. In particular, studying bioaccessibility requires direct access to gastrointestinal fluids which requires invasive procedures. The ethics of treating humans in vivo with Hg clearly prevent such studies from being done today, and instead we rely on information from limited and outdated human in vivo studies. Though, with advancements in Hg exposure science it may be possible to conduct detailed epidemiological surveys and when coupled with sophisticated biomarker analyses (e.g., see Sherman et al. [77]) there may be creative avenues to better resolve Hg bioavailability in populations.

Our models need to better understand what percentage of the ingested Hg reaches systemic circulation, and thus need more data on bioavailability. However, the vast majority of existing human-focused studies use in vitro models of bioaccessibility to estimate bioavailability. Bioaccessibility is a component of bioavailability, and has been proposed as a conservative estimate of bioavailability (effectively assuming 100% absorption and no metabolism; [13,48]). While conservative estimates are generally encouraged in public health contexts, when the exposure is coupled with significant health benefits associated with seafood consumption, overly conservative estimates of exposure may inadvertently harm public health.

Addressing the lack of in vitro/in vivo correlation in existing studies is key to demonstrating which in vitro methods best correspond to in vivo conditions. Some efforts have been made to compare extrapolated human population Hg exposure (based on measures of fish consumption and in vitro bioaccessibility studies) to estimates of “acceptable” exposure such as the Tolerable Daily Intake [57]. However, to our knowledge only one study [51] has attempted to integrate bioavailability data into a Hg exposure assessment study to see if this information could better improve the relationship between biomarkers of Hg (blood and hair) and fish consumption data. In this study, whether accounting for bioaccessibility alone or for bioaccessibility and absorption together, the ability to predict either Hg biomarker levels from survey-based fish consumption information did not improve [51]. There are several possible reasons for this: (1) concentrations of Hg in fish tissue were estimated for each type of fish from a FDA database rather than directly measured in subsamples of the fish that subjects from the cohort had actually eaten; (2) the uncertainty inherent in food frequency questionnaires which were used to collect the fish consumption data; and (3) the in vitro bioavailability portion of the study was only able to include the top 10 most-consumed fish, leaving a number of other fish for which bioavailability had to be assumed to be 100% (these “leftover” fish included some less-popularly consumed fish that have high Hg concentrations that may contribute a substantial portion of the exposure for the smaller number of individuals who consume them). Nonetheless, more studies are needed that consider bioavailability data in exposure assessments.

Another key challenge is a lack of standardized methods between different studies for assessing bioaccessibility in vitro; different studies use different enzyme concentrations, digestion lengths, and separation methods (for example, as reviewed in Hur et al. [78]). Notably, the choice of separation method has been demonstrated to influence the ultimate assessment of bioaccessibility from soil of a variety of metals, including Hg [79]. It is not yet known if such differences also apply for MeHg from seafood. For example, of the studies we reviewed 7 studies looked at 2 types of tuna, including Atlantic bluefin tuna (*Thunnus thynnus*) and unspecified tuna species (*Thunnus* spp.). In bluefin tuna, Hg(II) was 75% bioaccessible when raw, but when cooked it dropped to 5% (fried) and 25% (boiled). In unspecified tuna species, Hg(II) bioaccessibility ranged from 9%–78% (raw), 6%–39% (grilled), and was 48% (boiled), 18% (canned in olive oil), and 20% (canned in water) (Figure S1). MeHg bioaccessibility was not assessed for bluefin tuna, but for unspecified tuna it was 63%–84% (raw), 42%–44% (grilled), 57% (boiled), 18% (canned in olive oil), 29% (canned in water), 36%–99% (canned light), and 26%–76% (canned white) (Figure S2). Without a standardized method, it is difficult to determine whether this wide range of bioaccessibility reported for tuna is due to methodological differences, differences in location and source of the fish, species difference of the fish, or other factors.

Beyond the methodological aspects above, there is growing awareness of factors (e.g., nutrition, gut microbiome, and genetics) that may also contribute to variability in Hg bioavailability. Other nutritional factors that can influence Hg bioavailability are outlined in Table 2. The gut microbiome is implicated in bioavailability of Hg in rats [80], mice [81], and in vitro models [72], and given the great interest in the microbiome we anticipate seeing this incorporated into Hg bioavailability studies. The contribution of genetic variation to risk from Hg exposure has been reviewed elsewhere; of note for bioavailability, specific genetic polymorphisms in pathways such as the OAT, glutathione metabolism, metallothioneins, and selenoproteins, have already been associated with either higher or lower biomarker levels of MeHg or inorganic Hg [82].

In summary, for both fish and humans, a number of factors (e.g., Hg source, cooking methods, nutrients) are shown to affect Hg bioavailability. The summaries presented here challenge a widely-held assumption in the Hg risk assessment field, and along with further improvements in methodology for evaluating bioavailability, show potential to inform a more nuanced understanding of the risks due to Hg from consumption of various types of fish under certain conditions.

## Figures and Tables

**Table 1:** Effect of cooking or preparation treatment on Hg concentration, bioaccessibility, and bioavailability. Effects of cooking treatment expressed relative to raw fish; effects of skinning and trimming fat expressed relative to unskinned, untrimmed fish.

Fish Species	Cooking Treatment	Hg Concentration Effect	Weight Measurement	Hg Bioaccessibility Effect	Reference
Meagre (farmed; *Argyrosomus regius*)	Grilled	increased total Hg 33%; increased MeHg 25%	unclear; probably wet weight	Not studied	[55]
	Boiled	no change	unclear; probably wet weight	Not studied	
	Roasted	increased total Hg 19%; increased MeHg 19%	unclear; probably wet weight	Not studied	
Largemouth bass (*Micropterus salmoides*)	Deep fried (with breading)	increased total Hg 45%	wet weight	Not studied	[56]
	Deep fried (without breading)	increased total Hg 75%	wet weight	Not studied	
Spanish mackerel (*Scomberomorus macalatus*), cat shark (*Scyliorhinus* sp.), red tuna (*Thunnus thynnus*)	Fried	no change	dry weight	total Hg: 65% lower; MeHg: 85% lower	[54]
	Boiled	no change	dry weight	total Hg: 38% lower; MeHg: 54% lower	
Fresh swordfish (*Xiphias gladius*), frozen tope shark (*Galeorhinus galeus*), frozen bonito (*Sarda* sp.), fresh tuna (*Thunnus* sp.)	Hot plate/griddle	total Hg increased: swordfish (43%), tuna (32%), tope shark (22%), bonito (20%)	wet weight	total Hg in raw fish: 42% (13%–87%); total Hg in cooked fish: 26% (6%–49%)	[57]
	Hot plate/griddle	no change	dry weight	Not studied	
Sardine, hake, tuna	Fried	no change	wet weight	Not studied	[58]
	Grilled	no change	wet weight	Not studied	
Hake	Roasted	no change	wet weight	Not studied	
	Boiled	no change	wet weight	Not studied	
Striped bass	Baked	no change	dry weight	Not studied	[59]
	Broiled	no change	dry weight	Not studied	
	Fried	no change	dry weight	Not studied	
	Microwaved	no change	dry weight	Not studied	
	Poached	no change	dry weight	Not studied	
	Steamed	no change	dry weight	Not studied	
Brown trout (*Salmo trutta*)	Skinned, trimmed fat	increased total Hg 25% (male fish), 32% (female fish)	dry weight	Not studied	[60]

**Table 2:** Recent examples of nutrients/foods with effects on gut-relevant transport of MeHg.

Food or Nutrient	Hg Exposure	Model	Description of Interaction	Reference
Tea, coffee	1–4 μg/g dry weight Hg in fish (tuna, shark, mackerel)	in vitro digestion—bioaccessibility	Tea and coffee reduced total Hg bioaccessibility 10%–60%, depending on species	[54]
Corn starch	1–4 μg/g dry weight Hg in fish (tuna, shark, mackerel)	in vitro digestion—bioaccessibility	Corn starch reduced total Hg bioaccessibility by 20% (tuna only)	
Tropical Fruits	Hg in fish meals	Human, prospective study	Consumption of 1 fruit/day was associated with lower hair Hg than consumption of <1 fruit/day	[71]
Green tea extract	fish tissue	in vitro digestion—bioaccessibility	reduced Hg bioaccessibility by 82%–92%	[70]
Black tea extract	fish tissue	in vitro digestion—bioaccessibility	reduced Hg bioaccessibility by 88%–91%	
Soy protein	fish tissue	in vitro digestion—bioaccessibility	reduced Hg bioaccessibility by 44%–87%	
Grapefruit juice	fish tissue	in vitro digestion—bioaccessibility	no reduction of bioaccessible Hg	
Wheat bran	fish tissue	in vitro digestion—bioaccessibility	reduced Hg bioaccessibility by 84%	
OAT bran	fish tissue	in vitro digestion—bioaccessibility	reduced Hg bioaccessibility by 59%–85%	
Psyllium	fish tissue	in vitro digestion—bioaccessibility	reduced Hg bioaccessibility by 15%–31%	

## Supplementary Materials

The following are available online at www.mdpi.com/1660-4601/14/2/169/s1, Figure S1: Case study of MeHg bioaccessibility from tuna, Figure S2: Case study of Hg(II) bioaccessibility from tuna, Table S1: MeHg and Hg(II) assimilation efficiency for various fish species, Table S2: Key for studies for Figure 2, Table S3: Bioaccessibility of MeHg and Hg(II) to humans from various fish, Table S4: Key for studies for Figure 3 and Figure 4, Table S5: Absorption of MeHg and Hg(II) to humans from various fish.
