# Supplementary material for: A Review of Mercury Bioavailability in Humans and Fish
Source: Int J Environ Res Public Health. 2017 Feb 10;14(2):169. doi: 10.3390/ijerph14020169 (PMC5334723; doi:10.3390/ijerph14020169)
Supplement: Supplementary file 1 [file ijerph-14-00169-s001.pdf]

Supplementary Materials: A Review of Mercury Bioavailability in Humans and Fish

Mark A. Bradley, Benjamin D. Barst and Niladri Basu

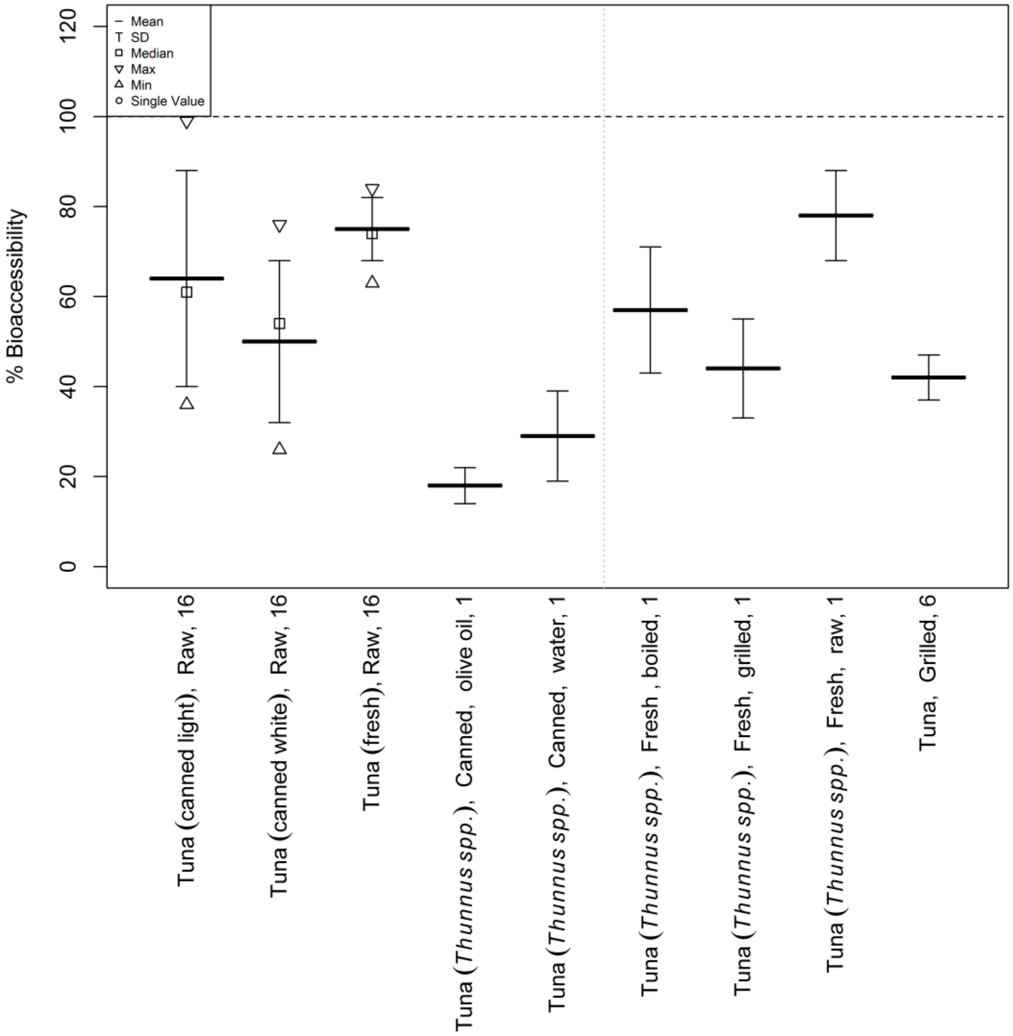

Figure S1. Case study of MeHg bioaccessibility from tuna.

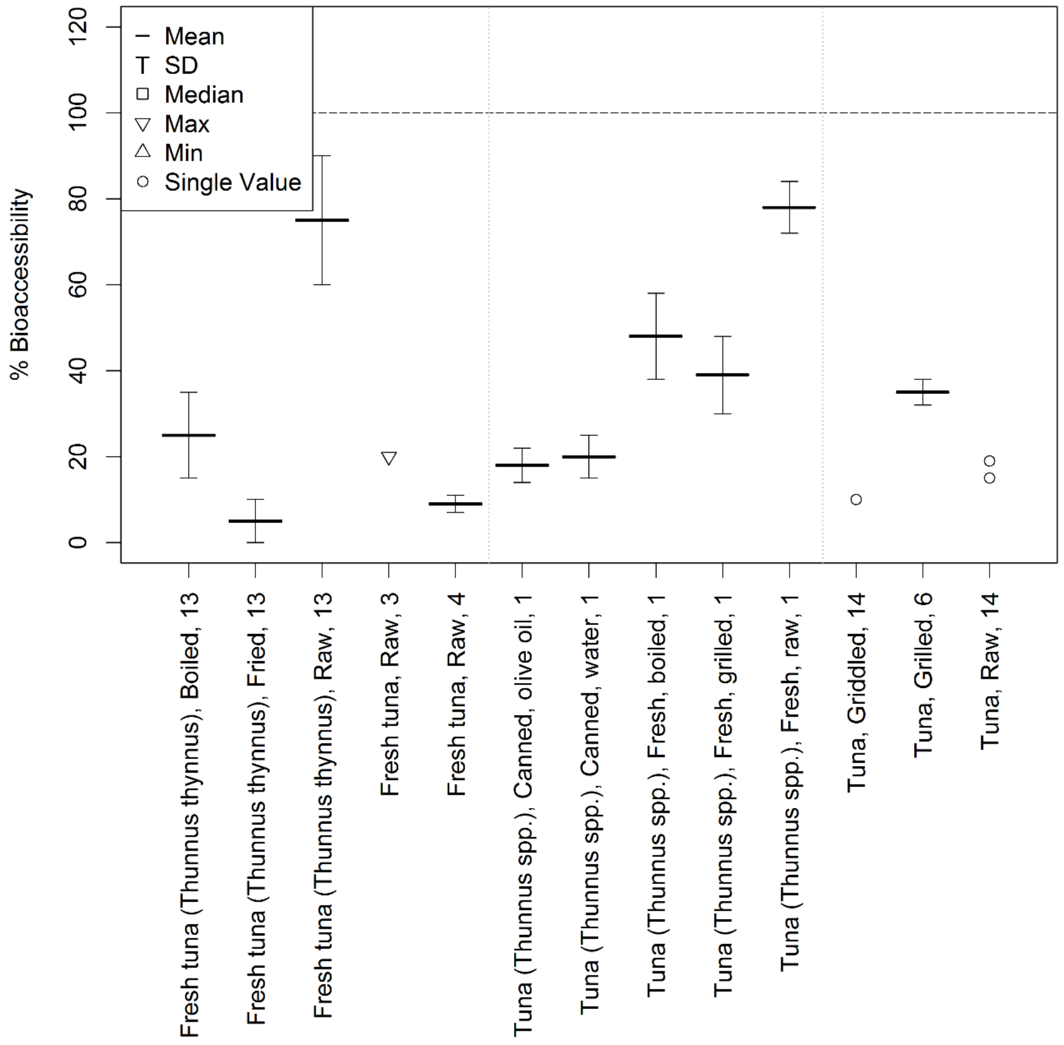

Figure S2. Case study of total Hg bioaccessibility from tuna.

Table S1. MeHg and Hg(II) assimilation efficiency for various fish species.

Study	Form(s) of Hg	Assimilation Efficiencies		Fish Species	Food Source	Duration of Experiment	Comments	Calculation of Assimilation Efficency (AE)
		Hg(II)	MeHg					
Pentreath 1976 [1]	^{203}Hg and ^{203}MeHg	9%	87%	Plaice *(Pleuronectes platessa)*	polychaete *(Nereis diversicolor)*	5 days		AEs were calculated as the percent of initial dose retained at the end of experiment.
	^{203}MeHg	NA	96%		starch pellets			
	^{203}MeHg	NA	93%		gelatine pellets			
	^{203}MeHg	NA	98%–100%		worm *(Arenicola marina)*			
	^{203}MeHg	NA	18%–26%		shrimp *(Crangon vulgaris)*			
	^{203}MeHg	NA	7%–42% *		mussel *(Mytilus edulis)*		* If the ^{203}Hg content of the shells were subtracted from the whole body values, the percentages of retained ^{203}Hg were 50%, 77%, and 42%. Therefore, these values are not presented in Figure 2.	
Phillips and Gregory 1979 [2]	Naturally contaminated fish (mean percent MeHg was 106% in whole-body homogenates of feeder fish)	NA	19%	Northern pike *(Esox lucius)*	common carp (*Cyprinus carpio*)	42 days		AE was calculated as percent of initial dose retained at the end of experiment.
Rodgers and Beamish 1982 [3]	^{203}MeHg	NA	70%–80%	Rainbow trout *(Salmo gairdneri)*	commercial trout food	28, 56, and 84 days	Low and intermediate treatments (0 and 25 µg Hg/g)	AEs were determined from the ratio of the intercept of the regression line (quantity of ^{203}MeHg in fish versus time after last meal to the quantitiy of ^{203}MeHg the fish were fed).
			<50%	Rainbow trout *(Salmo gairdneri)*		84 days	High dose treatment (75 µg Hg/g)	
Boudou and Ribeyre 1985 [4]	$HgCl_2$ and MeHgCl	23%	84%	Rainbow trout *(Salmo gairdneri)*	fry	30 days		AEs were calculated as percent of initial dose retained at the end of experiment.
Rouleau et al. 1998 [5]	^{203}MeHg	NA	88%	American plaice *(Hippoglossoides platessoides)*	food pellets	42 days		AE was calculated by extrapolating model curves to time 0 for two compartments.
Oliveira Ribeiro et al. 1999 [6]	^{203}Hg and ^{203}MeHg	~50% *	95%	Arctic char *(Salvelinus alpinus)*	food pellets	30 days	* Determined visually from figure.	AEs were calculated as percent of initial dose retained at the end of experiment.
Leaner and Mason 2002 [7]	MeHgCl	NA	61%	Channel catfish *(Ictalurus punctatus)*	bloodworms (*Glycera americana*)	36 h		AE calculated as: $(((MeHg_{Initial\ Dose}) - (MeHg_{Feces + Water}))/(MeHg_{Intial\ Dose})) \times 100$.

Table S1. *Cont.*

Study	Form(s) of Hg	Assimilation Efficiencies		Fish Species	Food Source	Duration of Experiment	Comments	Calculation of Assimilation Efficency (AE)
		Hg(II)	MeHg					
Wang and Wong 2003 [8]	^{203}Hg and $^{203}MeHg$	10%	90%	Sweetlips (*Plectorhinchus gibbosus*)	brine shrimp (*Artemia* sp.)	48 h		AEs were calculated as the percentage of ^{203}Hg or $^{203}MeHg$ retained in the fish at 24 h.
		27%	95%		copepods (*Acartia spinicauda*)			
		16%	56%		Silverside (*Atherion elymus*)			
Leaner and Mason 2004 [9]	MeHgCl	NA	90%	Sheepshead minnow (*Cyprindon variegatus*)	algae (*Tetraselmis* sp.)	35 days		AEs were calculated as: $(((MeHg_{Initial\ Dose}) - (MeHg_{Feces+Water}))/(MeHg_{Intial\ Dose})) \times 100$.
			92%		flake food			
Berntssen et al. 2004 [10]	$HgCl_2$ and MeHgCl	4%–6%	23%–41%	Atlantic salmon (*Salmo salar*)	prepared fish meal	4 months	6 groups per form of Hg	AEs were calculated as: (final total carcass Hg content) initial carcass Hg content) × 100/Hg(II) fed. Carcass = whole fish) gastro-intestinal tract.
Houck and Cech 2004 [11]	MeHgCl	NA	40%–61%	Sacramento blackfish (*Orthodon microlepidotus*)	commercial trout chow	35 days		AEs were was calculated as: Hg found in muscle/amount of Hg ingested.
			32%–43%			70 days		
Pickhardt et al. 2006 [12]	^{203}Hg and $^{203}MeHg$	42%–51%	90%–94%	Mosquitofish (*Gambusia affinis*)	*Daphnia pulex*	6 days		AEs were determined by regressing the radioactivity in each depurating fish against time. Depuration data for each replicate was analyzed separately to determine the y-intercept for AEs.
		9%–10%	86%–91%	Redear sunfish (*Lepomis microlophus*)	Amphipod (*Hyallela* sp.)			
Matthews and Fisher 2008 [13]	$^{203}MeHg$	NA	94%	Killifish (*Fundulus heteroclitus*)	*Daphnia pulex*	312 h		AEs were calculated by fitting a linear regression between 48 and 312 h.
			88%	Striped bass (*Morone saxatilis*)	killifish (*Fundulus heteroclitus*)			
Goto and Wallace 2009 [14]	$^{203}MeHg$	NA	52%	Mummichog (*Fundulus heteroclitus*)	grass shrimp (*Palaemonetes pugio*)	24 h		AEs were calculated as the ratio of radioactivity remaining at 24 h to the intial radioactivity.
			63%		amphipod (*Gammarus mucronatus*)			
			67%		clamworm (*Neanthes virens*)			
			74%		amphipod (*Leptocheirus plumulosus*)			
			89%		aquatic insect larva (*Chironomus dilutus*)			
			60%		juvenile sheepshead minnow (*Cyprinodon variegatus*)			

Table S1. *Cont.*

Study	Form(s) of Hg	Assimilation Efficiencies		Fish Species	Food Source	Duration of Experiment	Comments	Calculation of Assimilation Efficency (AE)
		Hg(II)	MeHg					
Dutton and Fisher 2010 [15]	^{203}Hg and ^{203}MeHg	8%	89%	Atlantic silverside (*Menidia menidia*)—Nova Scotia population	brine shrimp (*Artemia franciscana*)	6 days		AEs were determined by fitting an exponential regression between 48 h and 144 h depuration time points. AE = y-intercept.
		15%	82%	Atlantic silverside (*Menidia menidia*)—South Carolina population				
Dang and Wang 2010 [16]	^{203}Hg and ^{203}MeHg	41%	90%–94%	Jarbua terapon (*Terapon jarbua*)	brine shrimp (*Artemia salina*)	48 h		AEs were calculated as percentage of initial dose retained after 48 h of depuration.
		25%	90%–94%		clam (*Ruditapes philippinarum*)			
		43%	90%–94%		green mussel (*Perna viridis*)			
		23%	90%–94%		scallop (*Chlamys nobilis*)			
		36%	90%–94%		Jarbua terapon viscera (*Terapon jarbua*)			
Wang et al. 2010 [17]	^{203}Hg and ^{203}MeHg	15%–32%	90%–99%	Tilapia (*Oreochromis niloticus*)	Oligochaete (*Tubifex tubifex*)	48 h		AEs were calculated as the percentage of Hg retained in tilapia at 36 h.
		9%–18%	90%–99%		*Daphnia carinata*			
		4%–49%	84%–85%		algae (*Chlamydomonus reinhardtii*)			
Bowling et al. 2011 [18]	MeHgCl	NA	60%	Largemouth bass (*Micropterus salmoides*)	crayfish (*Procambarus clarkii*)	3 weeks		AEs were calculated as: Whole-body MeHg burden (mass)/total MeHg fed (mass) × 100%.
			79%		artificial fish food			
			94%		crayfish (*Procambarus clarkii*) (previously fed contaminated largemouth bass)			
Dutton and Fisher 2011 [19]	^{203}Hg and ^{203}MeHg	14%	92%	Killifish (*Fundulus heteroclitus*)	amphipod (*Leptocheirus plumulosus*)	9 days		AEs were determined by fitting an exponential regression between 48 h and 216 h depuration time points. AE = y-intercept.
		24%	92%		oligochaete (*Lumbriculus variegatus*)			
Dang and Wang 2011 [20]	^{203}Hg and ^{203}MeHg	38%	93%	Jarbua terapon (*Terapon jarbua*)	commercial food	48 h		AEs were calculated as percent of initial dose retained after 48 h.
Dang and Wang 2012 [21]	^{203}Hg and ^{203}MeHg	25%	91%	Black seabream (*Acanthopagrus schlegeli*)	brine shrimp (*Artemia salina*)	48 h		AEs were calculated as the ratio of radioactivity remaining at 48 h to the intial radioactivity.

Table S1. *Cont.*

Study	Form(s) of Hg	Assimilation Efficiencies		Fish Species	Food Source	Duration of Experiment	Comments	Calculation of Assimilation Efficency (AE)
		Hg(II)	MeHg					
Dutton and Fisher 2012 [22]	^{203}Hg and $^{203}MeHg$	2%–4%	10%–14%	Killifish (*Fundulus heteroclitus*)	sediment	9 days		AEs were determined by fitting an exponential regression between 48 h and 216 h depuration time points. AE = y-intercept.
		18%	82%		Algae (*Dunaliella tertiolecta*)			
Wang and Wang 2010 [23]	^{203}Hg and $^{203}MeHg$	27%–47%	90%–97%	Tilapia (*Oreochromis niloticus*)	brine shrimp (*Artemia salina*)	30 days		AEs were calculated as the percentage of Hg retained after 36 h.
Li et al. 2015 [24]	Hg in fish muscle	NA	98%	Goldfish (*Carassius auratus*)	fish meal prepared from naturally contaminated fish	45 days		AE was calculated as: ((MeHgFinal Mass in Fish) − (MeHgMass lost by elimination))/(MeHgTotal mass over uptake period).
Peng et al. 2016 [25]	$HgCl_2$ and MeHgCl	36%	68%	Rabbitfish (*Siganus canaliculatus*)	food pellets	39 h		AEs were calculated as the total fish radioactivity at 39 h divided by the initial fish radioactivity.

Table S2. Key for studies for Figure 2.

Study	Study Number for Figure 2
Pentreath 1976 [1]	1
Phillips and Gregory 1979[2]	2
Rodgers and Beamish 1982 [3]	3
Boudou and Ribeyre 1985 [4]	4
Rouleau et al. 1998 [5]	5
Oliveira Ribeiro et al. 1999 [6]	6
Leaner and Mason 2002 [7]	7
Wang and Wong 2003 [8]	8
Leaner and Mason 2004 [9]	9
Berntssen et al. 2004 [10]	10
Houck and Cech 2004 [11]	11
Pickhardt et al. 2006 [12]	12
Matthews and Fisher 2008 [13]	13
Goto and Wallace 2009 [14]	14
Dutton and Fisher 2010 [15]	15
Dang and Wang 2010 [16]	16
Wang et al. 2010 [17]	17
Bowling et al. 2011 [18]	18
Dutton and Fisher 2011[19]	19
Dang and Wang 2011 [20]	20
Dang and Wang 2012 [21]	21
Dutton and Fisher 2012 [22]	22
Wang and Wang 2010 [23]	23
Li et al. 2015 [24]	24
Peng et al. 2016 [25]	25

Table S3. Bioaccessibility of MeHg and total Hg to humans from various fish.

Seafood Type	Cooking/Storage Method	Bioaccessibility (%)					Sample Size	Study
		Data Type	MeHg		Total Hg			
Anchovy (fresh)	Raw	Single Values			77.00		1	Calatayud et al. 2012 [26]
					86.00		1	
Anglerfish (fresh)	Raw	Single Values			57.00		1	
					57.00		1	
Arctic char (*S. alpinus*)	Raw	Mean +/− SD			52.30	±1.6	6	Laird et al. 2009 [27]
Bartail flathead (*Platycephalus indicus*)	Raw	Mean +/− SD	46.50		47.20		10	Wang et al. 2013 [28]
Bigeye (*Priacanthus macracanthus*)	Raw	Mean +/− SD	43.20		39.80		33	
Bighead carp (*Aristichthys nobilis*)	Raw	Mean +/− SD	35.60		35.20		6	
Black Scabbard (*Aphanopus carbo*)	Raw	Mean +/− SD			46.23	±10	5	Maulvault et al. 2011 [29]
	Steamed				33.20	±15	5	
	Grilled				43.78	±15	5	
	Fried				23.51	±15	5	
Bleeker's grouper (*Epinephelus bleekeri*)	Raw	Mean +/− SD	53.20		40.60		10	Wang et al. 2013 [28]
blue shark (*Prionace glauca*)	Raw	Mean +/− SD	98.00	±5	94.00	±3	NA	Matos et al. 2015 [30]
	Steamed		59.00	±4	55.00	±5		
	Grilled		53.00	±3	52.00	±5		
Blue whiting (fresh)	Raw	Single Values			62.00		1	Calatayud et al. 2011 [26]
					68.00		1	
Bonito	Raw	Single Values			17.00		15	Torres-Escribano et al. 2011 [31]
					23.00		15	
					19.00		15	
	Grilled	Single Values			12.00		15	
					16.00		15	
					17.00		15	
Butter Clams (*Saxidomus giganteus*)	Raw	Mean +/− SD			50.00	±28.9	4	Laird et al. 2013 [32]
cat shark (*Scyliorhinus canicula*)	Raw	Mean +/− SD			80.00	±15	3	Ouedraogo & Amyot 2011 [33]
	Boiled				25.00	±15	3	
	Fried				20.00	±15	3	
Catfish (*Clarias fuscus*)	Raw	Mean +/− SD	56.10		48.70		21	Wang et al. 2013 [28]
Chinook Salmon (*Oncorhynchus tshawytscha*)	Raw	Mean +/− SD			49.00	±22.1	4	Laird et al. 2013 [32]
Clam (fresh)	Raw	Single Values			82.00		1	Calatayud et al. 2012 [26]

Table S3. *Cont.*

Seafood Type	Cooking/Storage Method	Bioaccessibility (%)					Sample Size	Study
		Data Type	MeHg		Total Hg			
Cod	Raw	Mean +/− SD	77.00	±25			6	Siedlikoswki et al. 2016 [34]
		Median	86.00					
		Min	35.00					
		Max	100.00					
Crab	Raw	Mean +/− SD	64.00	±27			6	Siedlikoswki et al. 2016 [34]
		Median	58.00					
		Min	32.00					
		Max	100.00					
Cuttlefish	Grilled	Mean +/− SD	<DL		77.00	±6	NA	Cano-Sancho et al. 2015 [35]
	fresh	Single Values			63.00		1	Calatayud et al. 2012 [26]
					65.00		1	
	frozen				54.00		1	
Golden threadfin bream (*Nemipterus virgatus*)	Raw	Mean +/− SD	59.20		43.60		15	Wang et al. 2013 [28]
Goldspotted rabbitfish (*Siganus punctatus*)	Raw	Mean +/− SD	35.00		33.60		36	
Grass carp (*Ctenopharyngodon idellus*)	Raw	Mean +/− SD	48.00		37.00		6	
Grey mullet (*Mulgil cephalus*)	Raw	Mean +/− SD	47.00		40.80		18	
Hake (fresh)	Raw, fresh	Single Values			81.00		1	Calatayud et al. 2012 [26]
					66.00		1	
	Raw, frozen				92.00		1	
					59.00		1	
Halibut	Raw	Mean +/− SD	93.00	±8			6	Siedlikoswki et al. 2016 [34]
		Median	95.00					
		Min	79.00					
		Max	100.00					
Mackerel	Grilled	Mean +/− SD	<DL		26.00	±7	NA	Cano-Sancho et al. 2015 [35]
Mandarin fish (*Siniperca kneri*)	Raw	Mean +/− SD	50.10		40.30		3	Wang et al. 2013 [28]
Meagre (*Argyrosomus regius*)	Raw	Mean +/− SD	100.00	±0.8	87.00	±2.4	15	Afonso et al. 2015 [36]
	Boiled		93.00	±0.94	91.00	±5.1	15	
	Grilled		64.00	±8.51	54.00	±14	21	
	Roasted		79.00	±0.64	83.00	±2.6	15	
Monkfish	Grilled	Mean +/− SD	<DL		61.00	±10	NA	Cano-Sancho et al. 2015 [35]
Mud carp (*Cirrhina molitorella*)	Raw	Mean +/− SD	42.40		34.10		15	Wang et al. 2013 [28]

Table S3. *Cont.*

Seafood Type	Cooking/Storage Method	Bioaccessibility (%)					Sample Size	Study
		Data Type	MeHg		Total Hg			
Mussel (fresh)	Raw	Single Values			38.00		1	Calatayud et al. 2012 [26]
					69.00		1	
	Steamed	Mean +/− SD	<DL		17.00	±6	NA	Cano-Sancho et al. 2015 [35]
Norway lobster (frozen)	Raw	Single Values			40.00		1	Calatayud et al. 2012 [26]
					81.00		1	
Orange-spotted grouper (*Epinephelus coioides*)	Raw	Mean +/− SD	57.90		51.70		9	Wang et al. 2013 [28]
Prawn (frozen)	Raw	Single Values			86.00		1	Calatayud et al. 2012 [26]
					75.00		1	
	Grilled	Mean +/− SD	<DL		21.00	±2	NA	Cano-Sancho et al. 2015 [35]
Rice field eel (*Monopterus albus*)	Raw	Mean +/− SD	38.40		39.20		14	Wang et al. 2013 [28]
Salmon (Spp. Unspecified)	Raw	Mean +/− SD	84.00	±17			6	Siedlikoswki et al. 2016 [34]
		Median	88.00					
		Min	60.00					
		Max	100.00					
		Single Values			102.00		1	Calatayud et al. 2012 [26]
					106.00		1	
Salmon (*Salmo salar*)	Raw	Mean +/− SD	<DL		89.80	±0.1	6	Costa et al. 2015 [37]
	Grilled				32.20	±0.4	6	
Salmon Eggs (NA)	Raw	Mean +/− SD			10.00	±7.6	6	Laird et al., 2013 [32]
Sardine	Raw	Mean +/− SD			11.00	±2	5	Cabañero et al. 2004 [38]
		Single Values			10.00		1	Cabañero et al. 2007 [39]
					50.00		1	Calatayud et al. 2012 [26]
					35.00		1	
	Grilled	Mean +/− SD	<DL		17.00	±10	NA	Cano-Sancho et al. 2015 [35]
Scallop	Raw	Mean +/− SD	100.00				6	Siedlikoswki et al. 2016 [34]
		Median	100.00					
Seabream	Grilled	Mean +/− SD	<DL		38.00	±3	NA	Cano-Sancho et al. 2015 [35]
Shrimp	Raw	Mean +/− SD	100.00				6	Siedlikoswki et al. 2016 [34]
		Median	100.00					
	Raw, frozen	Single Values			92.00		1	Calatayud et al. 2012 [26]
Small hake	Raw, fresh	Single Values			89.00		1	Calatayud et al. 2012 [26]
					58.00		1	
	Raw, frozen				105.00		1	
					98.00		1	

Table S3. *Cont.*

Seafood Type	Cooking/Storage Method	Bioaccessibility (%)					Sample Size	Study
		Data Type	MeHg		Total Hg			
Snakehead (*Channa asiatiea*)	Raw	Mean +/− SD	42.80		32.70		12	Wang et al. 2013 [28]
Snubnose pompano (*Trachinotus blochii*)	Raw	Mean +/− SD	38.80		36.90		9	
Sockeye Salmon (*Oncorhynchus nerka*)	Raw	Mean +/− SD			46.00	±21.3	4	Laird et al. 2013 [32]
Sole	Fresh	Single Values			67.00		1	Calatayud et al. 2012 [26]
	Frozen				105.00		1	
	Grilled	Mean +/− SD	<DL		50.00	±6	NA	Cano-Sancho et al. 2015 [35]
Spanish mackerel (*Scomberomorus maculatus*)	Raw	Mean +/− SD			80.00	±5	3	Ouedraogo & Amyot 2011 [33]
	Boiled				35.00	±5	3	
	Fried				20.00	±5	3	
Spotted snakehead (*Channa maculate*)	Raw	Mean +/− SD	49.50		36.60		10	Wang et al. 2013 [28]
Squid	Raw, frozen	Single Values			51.00		1	Calatayud et al. 2012 [26]
					54.00		1	
Swordfish	Raw	Mean +/− SD			17.00	±1	5	Cabañero et al. 2004 [38]
		Max	20.00		20.00		5	
		Single Values			87.00		3	Torres-Escribano 2011 [31]
					72.00		3	
					59.00		3	
					66.00		1	Calatayud et al. 2012 [26]
					42.00		1	
					75.00		1	
					55.00		1	
	frozen, fried	Mean +/− SD			45.00	±24	35	Jadan-Piedra et al. 2016 [40]
		Median			40.00			
		Min			14.00			
		Max			92.00			
	Grilled	Mean +/− SD	57.00	±2	45.00	±1	NA	Cano-Sancho et al. 2015 [35]
		Single Values			49.00		3	Torres-Escribano et al. 2011 [31]
					35.00		3	
					38.00		3	
Tilapia (Spp. Unspecified)	Raw	Mean +/− SD	80.00	±21			6	Siedlikoswki et al. 2016 [34]
		Median	85.00					
		Min	46.00					
		Max	100.00					
Tilapia (*Oreochromis mossambicus*)	Raw	Mean +/− SD	55.50		42.10		10	Wang et al. 2013 [28]
Tongue sole (*Cynoglossus robustus*)	Raw	Mean +/− SD	26.30		25.10		18	

Table S3. *Cont.*

Seafood Type	Cooking/Storage Method	Bioaccessibility (%)					Sample Size	Study
		Data Type	MeHg		Total Hg			
Tope Shark	Raw	Single Values			43.00		3	Torres-Escribano et al. 2011 [31]
					59.00		3	
					22.00		3	
	Grilled				47.00		3	
					34.00		3	
					36.00		3	
Tuna (*Thunnus thynnus*)	Raw	Mean +/− SD			75.00	±15	3	Ouedraogo & Amyot 2011 [33]
	Fried				5.00	±5	3	
	Boiled				25.00	±10	3	
Tuna (Spp. unspecified)	Raw	Mean +/− SD			9.00	±2	5	Cabañero et al. 2004 [38]
			78.00	±10	78.00	±6	5	Afonso et al. 2015 [41]
			75.00	±7			6	Siedlikoswki et al. 2016 [34]
		Median	74.00					
		Min	63.00					
		Max	84.00					
		Max			20.00		5	Cabanero et al. 2007 [39]
		Single Values			19.00		3	Torres-Escribano et al. 2011 [31]
					15.00		3	
					13.00		3	
	Grilled	Mean +/− SD	42.00	±5	35.00	±3	NA	Cano-Sancho et al. 2015 [35]
			44.00	±11	39.00	±9	5	Afonso et al. 2015 [41]
		Single Values			10.00		3	Torres-Escribano 2011 [31]
					10.00		3	
					6.00		3	
	Boiled	Mean +/− SD	57.00	±14	48.00	±10	5	Afonso et al. 2015 [41]
	Canned, olive oil	Mean +/− SD	18.00	±4	18.00	±4	25	
	Canned, water	Mean +/− SD	29.00	±10	20.00	±5	25	
	Canned Light	Mean +/− SD	64.00	±24			6	Siedlikoswki et al. 2016 [34]
		Median	61.00					
		Min	36.00					
		Max	99.00					
	Canned White	Mean +/− SD	50.00	±18			6	Siedlikoswki et al. 2016 [34]
		Median	54.00					
		Min	26.00					
		Max	76.00					

Table S3. *Cont.*

Seafood Type	Cooking/Storage Method	Bioaccessibility (%)				Sample Size	Study
		Data Type	MeHg		Total Hg		
Yellow croaker (*Pseudosciaena crocea*)	Raw	Mean +/− SD	19.50		22.10	15	Wang et al. 2013 [28]
Yellow seafin (*Acanthopagrus latus*)	Raw	Mean +/− SD	29.30		21.40	9	
Rabbitfish	Raw	Mean +/− SD	25.00	±5		5	He and Wang 2011 [42]
	Steamed		20.00	±5		5	
	Grilled		10.00	±1		5	
	Fried		5.00	±0.5		5	
Grouper	Raw	Mean +/− SD	65.00	±5		5	He and Wang 2011 [42]
	Steamed		17.00	±1		5	
	Grilled		7.00	±0.5		5	
	Fried		2.00	±0.5		5	
King Mackerel	Raw	Mean +/− SD	70.00			3	Shim et al. 2009 [43]

NA: not available; <DL: below detection limit.

Table S4. Key for studies for Figures 3 and 4.

Study	Study Number for Figures 3 and 4
Afonso et al. 2015 [36]	1
Afonso et al. 2015 [41]	2
Cabañero et al. 2007 [39]	3
Cabañero et al. 2004 [38]	4
Calatayud et al. 2012 [26]	5
Cano-Sancho et al. 2015 [35]	6
Costa et al. 2015 [37]	7
Jadan-Piedra et al. 2016 [40]	8
Laird et al. 2009 [27]	9
Laird et al. 2013 [32]	10
Matos et al. 2015 [30]	11
Maulvault et al. 2011 [29]	12
Ouedraogo & Amyot 2011 [33]	13
Torres-Escribano et al. 2011 [31]	14
Wang et al. 2013 [28]	15
Siedlikoswki et al. 2016 [34]	16
He and Wang 2011 [42]	17
Shim et al. 2009 [43]	18
Vazquez et al. 2013 [44]	19
Vazquez et al. 2015 [45]	20

Table S5. Absorption of MeHg and Hg(II) to humans from various fish.

Seafood Type, or Form of Hg	Cooking/Storage Method	Absorption (%)		Cells Used	Exposure Duration	Study
		MeHg	Total Hg			
cod	Raw	40.06	NA	Caco-2	2 h	Siedlikoswki et al. 2016 [34]
crab	Raw	29.02	NA	Caco-2	2 h	
halibut	Raw	49.94	NA	Caco-2	2 h	
Hg(II)		NA	55 [#]	Caco-2	2 h	Vazquez et al. 2013 [44]
		NA	52 [#]	Caco-2/HT29-MTX		
Hg(II) + Cysteine		NA	40 [#]	Caco-2	2 h	
		NA	50 [#]	Caco-2/HT29-MTX		
$Hg(NO_3)_2$		NA	49.4, 66.6 [#]	Caco-2	1 h	Vazquez et al. 2015 [45]
MeHg		79	NA [#]	Caco-2	2 h	Vazquez et al. 2013 [44]
		79	NA [#]	Caco-2/HT29-MTX		
MeHg + Cysteine		69	NA [#]	Caco-2	2 h	
MeHg + Cysteine		76	NA [#]	Caco-2/HT29-MTX	2 h	
salmon	Raw	61.54	NA	Caco-2	2 h	Siedlikoswki et al. 2016 [34]
scallop	Raw	42.86	NA	Caco-2	2 h	
Shrimp	Raw	60.53	NA	Caco-2	2 h	
Swordfish	Raw (fresh, frozen)	NA	49–69	Caco-2	2 h or 4 h	Calatayud et al. 2012 [26]
swordfish (*Xiphias gladius*)	raw	12.3–17.9	NA [#]	Caco-2	1 h	Vazquez et al. 2013 [44]
		11.7–17.7	NA [#]	Caco-2/HT29-MTX		
tilapia	raw	42.68	NA	Caco-2	2 h	Siedlikoswki et al. 2016 [34]
tuna (canned light)	Raw	47.72	NA	Caco-2	2 h	
tuna (canned white)	Raw	30.07	NA	Caco-2	2 h	
tuna (fresh)	Raw	54.11	NA	Caco-2	2 h	

[#] signifies that cellular retention and transport were measured separatelyin these studies, and were combined to calculate absorption.

Reference

1. Pentreath, R. The accumulation of mercury from food by the plaice, *Pleuronectes platessa* l. *J. Exp. Mar. Biol. Ecol.* **1976**, *25*, 51–65.
2. Phillips, G.; Gregory, R. Assimilation efficiency of dietary methylmercury by northern pike (*Esox lucius*). *J. Fish. Board Can.* **1979**, *36*, 1516–1519.
3. Rodgers, D.; Beamish, F. Dynamics of dietary methylmercury in rainbow trout, *Salmo gairdneri*. *Aquat. Toxicol.* **1982**, *2*, 271–290.
4. Boudou, A.; Ribeyre, F. Experimental study of trophic contamination of *Salmo gairdneri* by two mercury compounds—$HgCl_2$ and CH_3HgCl—Analysis at the organism and organ levels. *Water Air Soil Pollut.* **1985**, *26*, 137–148.
5. Rouleau, C.; Gobeil, C.; Tjälve, H. Pharmacokinetics and distribution of dietary tributyltin compared to those of methylmercury in the American plaice *Hippoglossoides platessoides*. *Ma. Ecol. Progr. Ser.* **1998**, *171*, 275–284.
6. Oliveira Ribeiro, C.; Rouleau, C.; Pelletier, E.; Audet, C.; Tjälve, H. Distribution kinetics of dietary methylmercury in the arctic charr (*Salvelinus alpinus*). *Environ. Sci. Technol.* **1999**, *33*, 902–907.
7. Leaner, J.J.; Mason, R.P. Factors controlling the bioavailability of ingested methylmercury to channel catfish and *Atlantic sturgeon*. *Environ. Sci. Technol.* **2002**, *36*, 5124–5129.
8. Wang, W.-X.; Wong, R.S. Bioaccumulation kinetics and exposure pathways of inorganic mercury and methylmercury in a marine fish, the sweetlips *Plectorhinchus gibbosus*. *Mar. Ecol. Progress Ser.* **2003**, *261*, 257–268.
9. Leaner, J.J.; Mason, R.P. Methylmercury uptake and distribution kinetics in sheepshead minnows, *Cyprinodon variegatus*, after exposure to CH_3Hg-spiked food. *Environ. Toxicol. Chem.* **2004**, *23*, 2138–2146.
10. Berntssen, M.; Hylland, K.; Julshamn, K.; Lundebye, A.K.; Waagbø, R. Maximum limits of organic and inorganic mercury in fish feed. *Aquac. Nutr.* **2004**, *10*, 83–97.
11. Houck, A.; Cech, J.J. Effects of dietary methylmercury on juvenile Sacramento blackfish bioenergetics. *Aquat. Toxicol.* **2004**, *69*, 107–123.
12. Pickhardt, P.C.; Stepanova, M.; Fisher, N.S. Contrasting uptake routes and tissue distributions of inorganic and methylmercury in mosquitofish (*Gambusia affinis*) and redear sunfish (*Lepomis microlophus*). *Environ. Toxicol. Chem.* **2006**, *25*, 2132–2142.
13. Mathews, T.; Fisher, N.S. Evaluating the trophic transfer of cadmium, polonium, and methylmercury in an estuarine food chain. *Environ. Toxicol. Chem.* **2008**, *27*, 1093–1101.
14. Goto, D.; Wallace, W.G. Influences of prey-and predator-dependent processes on cadmium and methylmercury trophic transfer to mummichogs (*Fundulus heteroclitus*). *Can. J. Fish. Aquat. Sci.* **2009**, *66*, 836–846.
15. Dutton, J.; Fisher, N.S. Intraspecific comparisons of metal bioaccumulation in the juvenile Atlantic silverside *Menidia menidia*. *Aquat. Biol.* **2010**, *10*, 211–226.
16. Dang, F.; Wang, W.-X. Subcellular controls of mercury trophic transfer to a marine fish. *Aquatic Toxicol.* **2010**, *99*, 500–506.
17. Wang, R.; Wong, M.-H.; Wang, W.-X. Mercury exposure in the freshwater tilapia *Oreochromis niloticus*. *Environ. Pollut.* **2010**, *158*, 2694–2701.
18. Bowling, A.M.; Hammerschmidt, C.R.; Oris, J.T. Necrophagy by a benthic omnivore influences biomagnification of methylmercury in fish. *Aquat. Toxicol.* **2011**, *102*, 134–141.
19. Dutton, J.; Fisher, N.S. Bioaccumulation of As, Cd, Cr, Hg(II), and MeHg in killifish (*Fundulus heteroclitus*) from amphipod and worm prey. *Sci. Total Environ.* **2011**, *409*, 3438–3447.
20. Dang, F.; Wang, W.-X. Antagonistic interaction of mercury and selenium in a marine fish is dependent on their chemical species. *Environ. Sci. Technol.* **2011**, *45*, 3116–3122.
21. Dang, F.; Wang, W.-X. Why mercury concentration increases with fish size? Biokinetic explanation. *Environ. Pollut.* **2012**, *163*, 192–198.
22. Dutton, J.; Fisher, N.S. Bioavailability of sediment-bound and algal metals to killifish *Fundulus heteroclitus*. *Aquat. Biol.* **2012**, *16*, 85–96.
23. Wang, R.; Wang, W.-X. Importance of speciation in understanding mercury bioaccumulation in tilapia controlled by salinity and dissolved organic matter. *Environ. Sci. Technol.* **2010**, *44*, 7964–7969.

24. Li, J.; Drouillard, K.G.; Branfireun, B.; Haffner, G.D. Comparison of the toxicokinetics and bioaccumulation potential of mercury and polychlorinated biphenyls in goldfish (*Carassius auratus*). *Environ. Sci. Technol.* **2015**, *49*, 11019–11027.
25. Peng, X.; Liu, F.; Wang, W.X. Organ-specific accumulation, transportation and elimination of methylmercury and inorganic mercury in a low Hg accumulating fish. *Environ. Toxicol. Chem.* **2016**, *35*, 2074–2083.
26. Calatayud, M.; Devesa, V.; Virseda, J.R.; Barbera, R.; Montoro, R.; Velez, D. Mercury and selenium in fish and shellfish: Occurrence, bioaccessibility and uptake by Caco-2 cells. *Food Chem. Toxicol. Int. J. Publ. Br. Ind. Biol. Res. Assoc.* **2012**, *50*, 2696–2702.
27. Laird, B.D.; Shade, C.; Gantner, N.; Chan, H.M.; Siciliano, S.D. Bioaccessibility of mercury from traditional northern country foods measured using an in vitro gastrointestinal model is independent of mercury concentration. *Sci. Total Environ.* **2009**, *407*, 6003–6008.
28. Wang, H.S.; Xu, W.F.; Chen, Z.J.; Cheng, Z.; Ge, L.C.; Man, Y.B.; Giesy, J.P.; Du, J.; Wong, C.K.; Wong, M.H. In vitro estimation of exposure of Hong Kong residents to mercury and methylmercury via consumption of market fishes. *J. Hazard. Mater.* **2013**, *248–249*, 387–393.
29. Maulvault, A.L.; Machado, R.; Afonso, C.; Lourenço, H.M.; Nunes, M.L.; Coelho, I.; Langerholc, T.; Marques, A. Bioaccessibility of Hg, Cd and As in cooked black scabbard fish and edible crab. *Food Chem. Toxicol.* **2011**, *49*, 2808–2815.
30. Matos, J.; Lourenco, H.M.; Brito, P.; Maulvault, A.L.; Martins, L.L.; Afonso, C. Influence of bioaccessibility of total mercury, methyl-mercury and selenium on the risk/benefit associated to the consumption of raw and cooked blue shark (*Prionace glauca*). *Environ. Res.* **2015**, *143*, 123–129.
31. Torres-Escribano, S.; Ruiz, A.; Barrios, L.; Vélez, D.; Montoro, R. Influence of mercury bioaccessibility on exposure assessment associated with consumption of cooked predatory fish in Spain. *J. Sci. Food Agric.* **2011**, *91*, 981–986.
32. Laird, B.D.; Chan, H.M. Bioaccessibility of metals in fish, shellfish, wild game, and seaweed harvested in British Columbia, Canada. *Food Chem. Toxicol. Int. J. Publ. Br. Ind. Biol. Res. Assoc.* **2013**, *58*, 381–387.
33. Ouedraogo, O.; Amyot, M. Effects of various cooking methods and food components on bioaccessibility of mercury from fish. *Environ. Res.* **2011**, *111*, 1064–1069.
34. Siedlikowski, M.; Bradley, M.; Kubow, S.; Goodrich, J.M.; Franzblau, A.; Basu, N. Bioaccessibility and bioavailability of methylmercury from seafood commonly consumed in North America: In vitro and epidemiological studies. *Environ. Res.* **2016**, *149*, 266–273.
35. Cano-Sancho, G.; Perello, G.; Maulvault, A.L.; Marques, A.; Nadal, M.; Domingo, J.L. Oral bioaccessibility of arsenic, mercury and methylmercury in marine species commercialized in Catalonia (Spain) and health risks for the consumers. *Food Chem. Toxicol.* **2015**, *86*, 34–40.
36. Afonso, C.; Costa, S.; Cardoso, C.; Bandarra, N.M.; Batista, I.; Coelho, I.; Castanheira, I.; Nunes, M.L. Evaluation of the risk/benefit associated to the consumption of raw and cooked farmed meagre based on the bioaccessibility of selenium, eicosapentaenoic acid and docosahexaenoic acid, total mercury, and methylmercury determined by an in vitro digestion model. *Food Chem.* **2015**, *170*, 249–256.
37. Costa, S.; Afonso, C.; Cardoso, C.; Batista, I.; Chaveiro, N.; Nunes, M.L.; Bandarra, N.M. Fatty acids, mercury, and methylmercury bioaccessibility in salmon (*Salmo salar*) using an in vitro model: Effect of culinary treatment. *Food Chem.* **2015**, *185*, 268–276.
38. Cabañero, A.I.; Madrid, Y.; Cámara, C. Selenium and mercury bioaccessibility in fish samples: An in vitro digestion method. *Anal. Chim. Acta* **2004**, *526*, 51–61.
39. Cabañero, A.I.; Madrid, Y.; Camara, C. Mercury-selenium species ratio in representative fish samples and their bioaccessibility by an in vitro digestion method. *Biol. Trace Element Res.* **2007**, *119*, 195–211.
40. Jadan-Piedra, C.; Clemente, M.J.; Devesa, V.; Velez, D. Influence of physiological gastrointestinal parameters on the bioaccessibility of mercury and selenium from swordfish. *J. Agric. Food Chem.* **2016**, *64*, 690–698.
41. Afonso, C.; Costa, S.; Cardoso, C.; Oliveira, R.; Lourenco, H.M.; Viula, A.; Batista, I.; Coelho, I.; Nunes, M.L. Benefits and risks associated with consumption of raw, cooked, and canned tuna (*Thunnus* spp.) based on the bioaccessibility of selenium and methylmercury. *Environ. Res.* **2015**, *143*, 130–137.
42. He, M.; Wang, W.X. Factors affecting the bioaccessibility of methylmercury in several marine fish species. *J. Agric. Food Chem.* **2011**, *59*, 7155–7162.

43. Shim, S.M.; Ferruzzi, M.G.; Kim, Y.C.; Janle, E.M.; Santerre, C.R. Impact of phytochemical-rich foods on bioaccessibility of mercury from fish. *Food Chem.* **2009**, *112*, 46–50.
44. Vazquez, M.; Calatayud, M.; Velez, D.; Devesa, V. Intestinal transport of methylmercury and inorganic mercury in various models of Caco-2 and HT29-MTX cells. *Toxicology* **2013**, *311*, 147–153.
45. Vázquez, M.; Devesa, V.; Vélez, D. Characterization of the intestinal absorption of inorganic mercury in Caco-2 cells. *Toxicol. In Vitro* **2015**, *29*, 93–102.

© 2017 by the authors; licensee MDPI, Basel, Switzerland. This article is an open access article distributed under the terms and conditions of the Creative Commons by Attribution (CC-BY) license (http://creativecommons.org/licenses/by/4.0/).
